# 24-Hour Glucose Profiles on Diets Varying in Protein Content and Glycemic Index

**DOI:** 10.3390/nu6083050

**Published:** 2014-08-04

**Authors:** Marleen A. van Baak

**Affiliations:** Department of Human Biology, NUTRIM School for Nutrition, Toxicology and Metabolism, Maastricht University Medical Centre+, P.O. Box 616, 6200MD Maastricht, The Netherlands; E-Mail: m.vanbaak@maastrichtuniversity.nl; Tel.: +31-43-3881630; Fax: +31-43-3670976

**Keywords:** glycemic index, glycemic load, mean 24-h glucose concentration, glucose variability, continuous glucose monitoring

## Abstract

Evidence is increasing that the postprandial state is an important factor contributing to the risk of chronic diseases. Not only mean glycemia, but also glycemic variability has been implicated in this effect. In this exploratory study, we measured 24-h glucose profiles in 25 overweight participants in a long-term diet intervention study (DIOGENES study on Diet, Obesity and Genes), which had been randomized to four different diet groups consuming diets varying in protein content and glycemic index. In addition, we compared 24-h glucose profiles in a more controlled fashion, where nine other subjects followed in random order the same four diets differing in carbohydrate content by 10 energy% and glycemic index by 20 units during three days. Meals were provided in the lab and had to be eaten at fixed times during the day. No differences in mean glucose concentration or glucose variability (SD) were found between diet groups in the DIOGENES study. In the more controlled lab study, mean 24-h glucose concentrations were also not different. Glucose variability (SD and CONGA1), however, was lower on the diet combining a lower carbohydrate content and GI compared to the diet combining a higher carbohydrate content and GI. These data suggest that diets with moderate differences in carbohydrate content and GI do not affect mean 24-h or daytime glucose concentrations, but may result in differences in the variability of the glucose level in healthy normal weight and overweight individuals.

## 1. Introduction

There is increasing evidence that the postprandial state is an important factor contributing to the risk of chronic diseases [1,2]. With respect to glucose homeostasis, not only mean glycemia, but also glycemic variability has been implicated in this effect. Both the carbohydrate content and the glycemic index of the diet affect postprandial glycemia and glycemic variability. The glycemic index (GI) of a diet is supposed to reflect the potential of a diet to raise blood glucose concentration in the postprandial periods. Dietary glycemic index is derived from the glycemic index of the individual carbohydrate-rich foods composing the diet and their relative contribution to the carbohydrate content of the diet [3]. Because the glucose response not only depends on the GI, but also on the total carbohydrate content of the diet, the concept of glycemic load, which is the product of GI and the total carbohydrate content of the diet, has been introduced [4].

Although the relevance of GI derived from standard GI tables for the prediction of postprandial glucose response in the context of habitual meals or diets is much debated [5,6,7], Wolever *et al.* (2006) have shown a reasonable correlation between meal GI and glucose area under the curve (AUC) and a good correlation between glycemic load and glucose AUC over 2 h after the test meal for a variety of meals containing different amounts of carbohydrates, fats and proteins [8]. Nevertheless, from the literature, it is still unclear whether diets differing in calculated glycemic index or glycemic load indeed lead to differences in 24-h glycemia, since inconsistent results have been reported [9,10,11]. Even less is known about the effect of diets differing in glycemic index or glycemic load on glycemic variability. The inconsistent results are likely to be due to difficulties inherent with the GI concept (e.g., uncertainties with respect to the exact GI values, which are influenced by, e.g., variety, ripeness, processing, cooking and cooling, but also by the presence of other macronutrients in mixed meals), to differences in the design of the studies (e.g., differences in GI of the diets, fully controlled or in daily living, short- or long-term, composition of the separate meals in a diet [12,13] or differences in the characteristics of the subjects studied (e.g., healthy, insulin resistant, diabetic)).

In this exploratory study, 24-h glucose profiles were measured in overweight subjects consuming different diets varying in protein content and glycemic index. They were participants in a long-term diet intervention study (DIOGENES study on Diet, Obesity and Genes). In addition these diets were compared in a more controlled fashion where subjects were provided with meals that they had to eat at fixed times during the day. It was hypothesized that mean 24-h and, especially, daytime glucose concentration would differ among the four diets, with the highest levels on the diet combining a high GI with a high carbohydrate content, *i.e.*, the low protein-high GI (LP/HGI) diet, and the lowest on the diet combining a low carbohydrate content with a low GI, *i.e.*, the high protein-low GI (HP/LGI) diet, and that these effects would be most pronounced in the controlled situation. Continuous glucose monitoring also allows quantification of glucose variability over 24 h. The variability of glucose concentrations was expected to be larger on the LP/HGI diet than on the other diets, since this diet combined a high carbohydrate intake with a high GI of the carbohydrates consumed.

## 2. Subjects and Methods

Two studies were performed. The first one was a field study in the context of the DIOGENES study, a randomized controlled trial on the effects of diets differing in protein content and glycemic index on weight maintenance after weight loss. The design and main outcomes of the DIOGENES study have been reported earlier [14,15,16]. The second one was a lab study, in which similar diets were tested under more controlled conditions. During both studies, glucose concentrations were monitored during 72 h by continuous glucose monitoring (CGM).

### 2.1. Study Design

In the field study, subjects kept 3-day dietary records from which dietary intake was calculated. Subjects performed their normal habitual activities, both with respect to food consumption pattern and physical activities during the three measurement days. In the lab study, subjects followed each of the four diets during three days in a randomized cross-over design with a wash-out period of minimally four days between diets. Subjects were provided all foods for the 72 h by the investigators. They ate their breakfast, lunch and evening meal in the lab and were provided with a morning, afternoon and evening snack to consume at home or work. All meals, drinks and snacks were consumed at prescribed times. Apart from the meals in the lab, subjects were free to carry out their normal daily activities during the three measurement days. However, subjects were instructed to standardize their physical activities with respect to type, intensity and time on the three measurement days over the four diet periods.

Protocols of both studies were approved by the Medical Ethical Committee of Maastricht University. All subjects were informed about the nature, potential risks and discomfort associated with the study and gave written informed consent before the start of the study.

### 2.2. Subjects

In the field study, 25 non-diabetic, healthy, overweight and obese subjects were included. Subjects were recruited from participants of the European DIOGENES study in the Maastricht center. They had been on *ad libitum* weight maintenance diets differing in protein content and glycemic index for between 24 and 48 weeks (mean 29.5 weeks, no differences in duration among diet groups) after an 8-week weight loss period in which they had lost at least 8% of their initial body weight. In the lab study, 9 other non-diabetic, healthy, normal weight or overweight subjects, who did not participate in the DIOGENES study, were included.

### 2.3. Diets

In the field study, subjects had been randomized into 5 different diets: lower protein (LP)/lower GI (LGI), higher protein (HP)/LGI, LP/higher GI (HGI), HP/HGI and a control diet according to national recommendations for a healthy diet. Subjects on the control diet were not included in this study. The intended difference in protein content of the LP and HP diets was 10%–12% of energy intake; the intended difference in glycemic index between the LGI and HGI diets was 15. All diets were reduced in fat (<30 energy%). Subjects kept a food diary on the days of glucose monitoring.

The four diets in the lab study had comparable differences in macronutrient composition to those in the field study, but the GI difference between the LGI and HGI diets was slightly larger (20 units). The energy content of these diets was derived from basal metabolic rate (estimated by means of the Schofield equation [17]) multiplied with a factor for the physical activity level of each subject (1.4 to 1.6). The composition of these diets is shown in Table 1. Each food in the diets was assigned a GI value based on the DIOGENES GI database [10]. Dietary GI was calculated as:

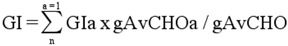
(1)
*n* was the number of carbohydrate-containing foods, GIa was the GI of the a^th^ food, gAvCHOa was grams of available carbohydrates in the a^th^ food and gAvCHO was grams of available carbohydrate in the entire diet.

### 2.4. Continuous Glucose Measurement

The sensor of a continuous glucose monitoring system (MiniMed CGMS Gold, Medtronics, Heerlen, The Netherlands) was inserted into the subcutaneous abdominal fat of the subjects in the afternoon before the first test day. The system was calibrated four times per day (before breakfast, before lunch, before dinner and before going to bed) with blood glucose concentrations in capillary blood obtained from finger pricks (One Touch Ultra, LifeScan, Tilburg, The Netherlands) by the subjects. Glucose concentrations were measured and stored every 5 min during the whole period the device was worn by the subjects. After the device had been removed (in the morning of the fifth day), the data were downloaded to a PC. The last two 24-h periods were used for further analysis. This was done to minimize a potential effect of the unstandardized diet on the day before the test days in the lab study. In order to keep the number of days in the field and lab study comparable, this was also done in the field study.

### 2.5. Data Analysis

Results are presented as means ± SD. In the field study, average glucose concentration and its standard deviation were calculated over 48 h in each subject. In addition, average daytime (from 6 AM to 10 PM) and nighttime (from 10 PM to 6 AM) concentrations and their SD were calculated in each subject. Subsequently, these data were averaged for each diet group. Differences between diets were analyzed by one-way ANOVA. In the lab study, individual glucose profiles and the SDs of the two 24-h periods were averaged. Continuous overall net glycemic action values with a time period of 60 min (CONGA1) over 24 h were calculated, as well [18]. Repeated measurement ANOVAs were used to compare diets. We *a priori* hypothesized that glucose concentration and its variability would be highest on the LP/HGI diet, and therefore, we performed pairwise comparisons between the LP/HGI diet and the other diets by Dunnett’s tests. A *p*-value < 0.05 is considered statistically significant.

**Table 1:** Composition of the diets in the lab study (energy content: 2000 kcal). HP, high protein; LP, low protein; LGI, low glycemic index (GI); HGI, high GI.

Meal	LP/LGI	LP/HGI	HP/LGI	HP/HGI
Breakfast	Rye bread (103 g)	Wheat bread (82.6 g)	Rye bread (100 g)	Wheat bread (85.7 g)
	Margarine spread 60% fat (12.9 g)	Margarine spread 60% fat (11.8 g)	Margarine spread 60% fat (12.5 g)	Margarine spread 35% fat (12.2 g)
	Soft cheese (25.8 g)	Soft cheese (23.6 g)	Ham (25 g)	Ham (24.5 g)
	Apple spread (19.3 g)	Apple spread (17.7 g)	Cheese 30^+^ (25 g)	Cheese 30^+^ (24.5 g)
	Water	Water	Milk 28% fat (187.6 g)	Milk 28% fat (183.7 g)
Morning snack	Apple (193.2 g)	Banana (153.5 g)	Apple (187.6 g)	Banana (159.2 g)
Lunch	Rye bread (154.6 g)	Wheat bread (124 g)	Rye bread (150 g)	Wheat bread (126.6 g)
	Margarine spread 60% fat (19.3 g)	Margarine spread 60% fat (17.7 g)	Margarine spread 60% fat (18.8 g)	Margarine spread 35% fat (18.4 g)
	Jam (19.3 g)	Jam (17.7 g)	Ham (25 g)	Ham (24.5 g)
	Cheese 30^+^ (12.9 g)	Cheese 30^+^ (11.8 g)	Cheese 30^+^ (50 g)	Cheese 30^+^ (49 g)
	Soft cheese (25.8 g)	Sandwich spread (17.7 g)		
	Chocolate milk 193.2 g)	Chocolate milk (177.1 g)	Milk 28% fat (187.6 g)	Milk 28% fat (183.7 g)
Afternoon snack	Pear (206.1 g)	Banana (153.5 g)	Pear (200 g)	Banana (159.2 g)
Dinner	Macaroni pasta (257.6 g)	Fried potato slices (236.1 g)	Macaroni pasta (250 g)	Fried potato slices (245 g)
	Broccoli (257.6 g)	Carrots (236.1 g)	Broccoli (250 g)	Carrots (245 g)
	Fried chicken filet (64.4 g)	Fried chicken filet (59.0 g)	Fried chicken filet (125 g)	Fried chicken filet (122.5 g)
	Fruit yoghurt 1.5% fat (257.6 g)	Fruit yoghurt 1.5% fat (236.1 g)	Fruit yoghurt 0% fat (250 g)	Fruit yoghurt 0% fat (245 g)
	Mushroom sauce (128.8 g)	Yoghonaise (17.7 g)	Mushroom sauce (125 g)	Yoghonaise (18.4 g)
Evening snack		Kiwi (106.3 g)		Kiwi (110.2 g)
	Liga Continue cookie (32.2 g)	Liga Bastogne cookie (23.6 g)	Cheese 30^+^ (25 g)	Cheese 30^+^ (24.5 g)

## 3. Results

### 3.1. Field Study

Characteristics of the subjects in the field study are shown in Table 2. There were no statistically significant differences between the diet groups in these characteristics (data not shown). Total weight loss from the start of the DIOGENES study was 8.8 ± 8.4 kg, and weight regain since the start of the randomized diet intervention was 1.6 ± 7.3 kg, with no significant differences between the diet groups (data not shown). Analysis of the three-day dietary records showed the expected differences in carbohydrate content and glycemic index between the diet groups, although contrasts were smaller than intended (Table 3). There were no significant differences in mean 24-h, daytime or nighttime glucose concentrations between the diet groups, nor did the SDs differ significantly (Table 4). *Post hoc* testing did not reveal differences between the LP/HGI diet and any of the other diets for the glucose parameters studied. Adjustment for BMI, HOMA-IR (Homeostatic Model Assessment of Insulin Resistance) index, minutes of exercise performed by the subjects, total weight loss or weight regain did not change this outcome (data not shown).

**Table 2:** Subject characteristics (mean ± SD) in the field and the lab study. HOMA-IR: Homeostatic Model Assessment of Insulin Resistance.

Subject Characteristic	Field Study (*N* = 25)	Lab Study (*N* = 9)
Age (year)	44.3 ± 4.9	30.3 ± 11.7
Gender (M, F)	9 M, 16 F	2 M, 7 F
Height (m)	1.69 ± 0.08	1.71 ± 0.08
Body weight (kg)	83.5 ± 11.5	72.0 ± 15.5
BMI (kg/m^2^)	29.5 ± 2.9	24.5 ± 3.9
Fasting glucose concentration (mmol/L)	5.1 ± 0.6	5.0 ± 0.3
Fasting insulin concentration (mU/L)	10.2 ± 5.3	10.4 ± 1.7
HOMA-IR	2.40 ± 1.51	2.30 ± 0.25

**Table 3:** Self-reported dietary macronutrient composition in the field study (**a**) and macronutrient composition of the diet in the lab study (**b**).

Diet Group	Fat (% of Energy)	Protein (% of Energy)	CHO (% of Energy)	GI
**a.**				
LP/LGI	22 ± 9	13 ± 3	64 ± 9	54 ± 2
LP/HGI	29 ± 10	16 ± 5	51 ± 11	64 ± 4
HP/LGI	31 ± 4	20 ± 4	48 ± 2	53 ± 3
HP/HGI	31 ± 5	23 ± 4	45 ± 3	61 ± 4
One-way ANOVA	*p* = 0.294	*p* = 0.003	*p* = 0.002	*p* < 0.001
**b.**				
LP/LGI	27	15	58	40
LP/HGI	29	14	57	60
HP/LGI	27	26	47	40
HP/HGI	28	26	46	60

**Table 4:** Parameters of glucose homeostasis (mean ± SD) on the different diets in the field study.

Parameter of Glucose Homeostasis	LP/LGI (*N* = 3)	LP/HGI (*N* = 7)	HP/LGI (*N* = 6)	HP/HGI (*N* = 9)	*p*-Value One-Way ANOVA
24-h glucose (mmol/L)	5.17 ± 0.50	5.54 ± 0.47	5.15 ± 0.41	5.49 ± 0.59	0.444
SD 24-h glucose (mmol/L)	0.93 ± 0.28	0.70 ± 0.17	0.64 ± 0.25	0.69 ± 0.21	0.507
Daytime glucose (mmol/L)	5.16 ± 0.60	5.49 ± 0.51	5.18 ± 0.38	5.51 ± 0.56	0.507
SD daytime glucose (mmol/L)	1.00 ± 0.32	0.72 ± 0.22	0.66 ± 0.31	0.68 ± 0.17	0.195
Nighttime glucose (mmol/L)	5.19 ± 0.32	5.65 ± 0.44	5.10 ± 0.54	5.45 ± 0.69	0.351
SD nighttime glucose (mmol/L)	0.72 ± 0.30	0.58 ± 0.16	0.50 ± 0.19	0.45 ± 0.27	0.321

No significant differences between LP/HGI and any other diet group (Dunnett’s test).

### 3.2. Lab Study

**Figure 1:**
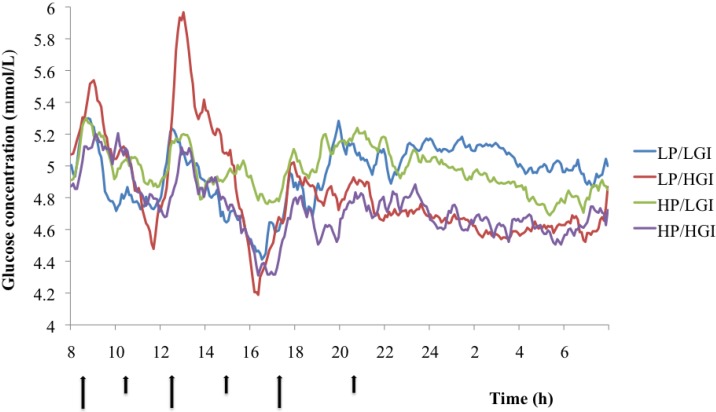
Mean glucose concentration over 48 h on the different diets in the lab study. Arrows at the horizontal axis reflect meal and snack times (large arrows = meals, small arrows = snacks).

**Figure 2:**
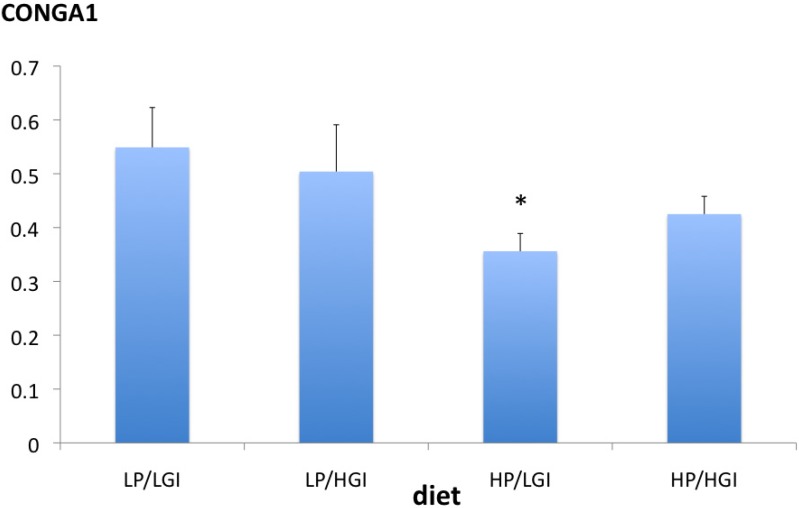
Continuous overlapping net glycemic action over 1 h periods (CONGA1) values (mean, SE) over 24 h on the different diets in the lab study. * *p* = 0.03 *vs.* LP/LGI (Dunnett’s test).

Characteristics of the subjects in the lab study are presented in Table 2, and diet compositions are shown in Table 3. The GI contrast between the diets was larger than in the field study; the protein contrast was comparable. Glucose parameters (mean over 24-h, daytime) did not differ significantly between the LP/HGI diet and any other diet (Figure 1 and Table 5). However, the mean nighttime glucose concentration was significantly higher on the LP/LGI diet than on the LP/HGI diet (Dunnett’s test, *p* < 0.02) (Table 5). Daytime glucose variability, as reflected by SD, was significantly different among diets (one-way ANOVA, *p* < 0.05). Moreover, 24-h and daytime glucose variability were significantly higher on the LP/HGI diet than on the HP/LGI diet (Dunnett’s test, both *p* < 0.02) (Table 5). Analysis of the CONGA1 (continuous overlapping net glycemic action over a 1h period) values confirmed the higher variability of glucose concentrations on the LP/HGI diet compared to the HP/LGI diet (Dunnett’s test, *p* = 0.03) (Figure 2).

**Table 5:** Parameters of glucose homeostasis (mean ± SD) on the different diets in the lab study.

Parameter of Glucose Homeostasis	LP/LGI	LP/HGI	HP/LGI	HP/HGI	*p*-Value One-Way ANOVA
24-h glucose (mmol/L)	4.95 ± 0.40	4.83 ± 0.29	4.97 ± 0.39	4.73 ± 0.42	0.312
SD 24-h glucose (mmol/L)	0.42 ± 0.16	0.45 ± 0.20	0.28 ± 0.09 ^#^	0.37 ± 0.12	0.086
Daytime glucose (mmol/L)	4.89 ± 0.47	4.92 ± 0.25	4.99 ± 0.37	4.76 ± 0.35	0.402
SD daytime glucose (mmol/L)	0.44 ± 0.18	0.50 ± 0.23	0.30 ± 0.10 ^#^	0.36 ± 0.12	0.035
Nighttime glucose (mmol/L)	5.07± 0.36 ^#^	4.64 ± 0.39	4.93 ± 0.44	4.67 ± 0.62	0.076
SD nighttime glucose (mmol/L)	0.20 ± 0.09	0.17 ± 0.06	0.20 ± 0.10	0.24 ± 0.13	0.494

^#^ *p* < 0.02 compared to LP/HGI (Dunnett’s test).

## 4. Discussions

In this study, the effects of dietary carbohydrate content and glycemic index on 24-h glucose concentrations were investigated under two conditions: in a field study, where food intake was not controlled with respect to timing, food choice, meal size and composition, and in a lab study, where these aspects were controlled. It was hypothesized that glucose concentrations would be highest on the reference diet (LP/HGI) compared to all other diets and especially compared to the HP/LGI diet. The data do not support this hypothesis. On the other hand, support for our other hypothesis regarding glucose variability was found: both 24-h and daytime variability of glucose concentrations were lower on the HP/LGI diet than on the LP/HGI diet.

A recent review discussed the role of postprandial glycemia and glycemic variability in the maintenance of optimal glycemic control, the prevention of obesity, diabetes and cardiovascular disease and for the optimization of exercise and cognitive performance [2]. The conclusion was that, although there is mechanistic evidence linking postprandial glycemia or glycemic variability to health and disease conditions, good quality intervention studies with well-defined differences in postprandial glycemia or glycemic variability are currently scarce. Manipulating the glycemic index and/or carbohydrate content of the diet is one potential way to create variation in postprandial glycemia and glycemic variability. The results of our study show that moderate differences in glycemic index (8–10 GI units) and carbohydrate content (6%–16% of total energy intake) of a habitual *ad libitum* diet in a real life setting do not result in measurable differences in mean glucose concentration or glucose variability in overweight and obese subjects who had been following these diets for at least six months, suggesting more extreme differences and possibly less meal-to-meal and day-to-day variation are required to be able to detect differences in glycemic responses. Because the carbohydrate content of the diets where we expected the largest contrast in glycemic response (LP/HGI and HP/LGI) turned out not to differ significantly in carbohydrate content as intended, the contrast between the two diets was probably too small. The lab study, which created a larger difference in GI (20 GI units) in combination with a comparable difference in carbohydrate content (10% of total energy intake) between the two diets showed significantly more pronounced glucose variability on the LP/HGI diet as compared to the HP/LGI diet. Whether the adaptation of these glucose responses to long-term dietary changes occurs is unknown. It therefore cannot be excluded that such an adaptation has also contributed to the difference between the (long-term) field study and the (acute) lab study.

Results of previous studies on the effect of dietary carbohydrate content and glycemic index on 24-h glucose profiles have been inconsistent. Brynes *et al.* (2005) compared 24-h glucose profiles in 10 healthy adults during their habitual diet and after consumption of a low GI diet with a comparable total carbohydrate content for at least seven days [11]. The difference in GI between the habitual diet and the LGI diet was eight units (*p* < 0.001). Subjects were requested to keep activity patterns constant and to refrain from alcohol consumption while wearing the glucose monitors. Mean 24-h glucose concentration, 24-h AUC, overnight glucose concentration and glucose concentration at 6 AM were all significantly lowered on the LGI diet. Fabricatore *et al.* (2011) measured 24-h glucose profiles for three days in 26 overweight or obese adults with type 2 diabetes consuming their habitual diet. Dietary GI varied between 50 and 80 and was significantly correlated with the glucose AUC (*r* = 0.38, *p* ≤ 0.05). The correlation with glycemic load was not statistically significant (*r* = 0.26, *p* ≤ 0.10) [19]. In our study, the correlation between dietary GI and glucose AUC in the field study was not significant (*r* = 0.22, *p* = 0.32); GI varied between 49 and 68. Therefore, a larger GI contrast may be needed to be able to detect such a correlation. Aston *et al.* (2010) compared diets differing from 14 to 15 GI units with comparable carbohydrate content in 12 healthy, overweight women and found no differences in 24-h glucose profiles, neither in the home situation nor in a controlled day in the lab [10]. Subjects had been on the diets for four days before the glucose monitoring was started. Henry *et al.* (2006) tested the acute effects of high GI bread (GI = 54) and low GI bread (GI = 49) during breakfast and lunch in for the rest identical diets on 24-h glucose concentration in 10 healthy subjects. No statistically significant effects on 24-h, daytime or nighttime glucose concentrations were found [9]. In none of the above studies was glycemic variability addressed.

A significantly higher nighttime glucose concentration on the LP/LGI diet compared to the LP/HGI diet was found in the lab study. This is an unexpected finding, since most studies reported a lower nighttime glucose concentration on low glycemic index diets [10,11], and the results of the field study did not show significant differences in nighttime glucose concentrations between the LGI and HGI groups. In the lab study, nighttime glucose concentration also tended to be higher on the HP/LGI diet compared to the HP/HGI diet (*p* = 0.08). If these findings are not due to chance, it would suggest that nighttime hepatic insulin sensitivity and/or nighttime insulin levels are reduced on the LGI diets compared to the HGI diets.

The limitations of this study are the small number of subjects in the two sub-studies with the risk of not being able to detect differences that may be there. Another limitation is that the field and the lab study are not directly comparable, because of differences in GI and carbohydrate contrast between the diets and differences in subject population.

## 5. Conclusions

Moderate differences in dietary GI that can be maintained long-term in the context of a habitual diet, as in the DIOGENES study, do not result in differences in mean glucose concentrations or glucose variability. Under more controlled conditions with a slightly larger GI contrast, mean 24-h glucose concentrations were also not different. Glucose variability, however, was lower on the diet combining the lower carbohydrate content with the lower GI compared to the diet with the higher carbohydrate content and the higher GI. These data suggest that diets with moderate differences in carbohydrate content and GI do not affect 24-h or daytime glucose concentrations, but may result in differences in the variability of the glucose level in healthy normal weight and overweight subjects.
